# Gut dysbiosis in Thai intrahepatic cholangiocarcinoma and hepatocellular carcinoma

**DOI:** 10.1038/s41598-023-38307-2

**Published:** 2023-07-14

**Authors:** Yotsawat Pomyen, Jittiporn Chaisaingmongkol, Siritida Rabibhadana, Benjarath Pupacdi, Donlaporn Sripan, Chidchanok Chornkrathok, Anuradha Budhu, Vajarabhongsa Budhisawasdi, Nirush Lertprasertsuke, Anon Chotirosniramit, Chawalit Pairojkul, Chirayu U. Auewarakul, Teerapat Ungtrakul, Thaniya Sricharunrat, Kannikar Phornphutkul, Suleeporn Sangrajang, Christopher A. Loffredo, Curtis C. Harris, Chulabhorn Mahidol, Xin Wei Wang, Mathuros Ruchirawat, TIGER-LC Consortium

**Affiliations:** 1 Translational Research Unit, Chulabhorn Research Institute, Bangkok, 10210 Thailand; 2 Laboratory of Chemical Carcinogenesis, Chulabhorn Research Institute, Bangkok, 10210 Thailand; 3 Center of Excellence on Environmental Health and Toxicology (EHT), OPS, MHESI, Bangkok, Thailand; 4 Liver Cancer Program, Center for Cancer Research, National Cancer Institute, Bethesda, MD 20892 USA; 5 Laboratory of Human Carcinogenesis, Center for Cancer Research, National Cancer Institute, Bethesda, MD 20892 USA; 6 Faculty of Medicine, Khon Kaen University, Khon Kaen, 40002 Thailand; 7 Faculty of Medicine, Chiang Mai University, Chiang Mai, 50200 Thailand; 8 Princess Srisavangavadhana College of Medicine, Chulabhorn Royal Academy, Bangkok, 10210 Thailand; 9 Chulabhorn Hospital, Chulabhorn Royal Academy, Bangkok, 10210 Thailand; 10 Rajavej Hospital, Chiang Mai, 50000 Thailand; 11 National Cancer Institute, Bangkok, 10400 Thailand; 12 Georgetown University Medical Center, Washington, DC 20057 USA; 13 Laboratory of Human Carcinogenesis, Center for Cancer Research, National Cancer Institute, Bethesda, MD 20892 USA

**Keywords:** Metagenomics, Gastrointestinal cancer

## Abstract

Primary liver cancer (PLC), which includes intrahepatic cholangiocarcinoma (iCCA) and hepatocellular carcinoma (HCC), has the highest incidence of all cancer types in Thailand. Known etiological factors, such as viral hepatitis and chronic liver disease do not fully account for the country’s unusually high incidence. However, the gut-liver axis, which contributes to carcinogenesis and disease progression, is influenced by the gut microbiome. To investigate this relationship, fecal matter from 44 Thai PLC patients and 76 healthy controls were subjected to whole-genome metagenomic shotgun sequencing and then analyzed by marker gene-based and assembly based methods. Results revealed greater gut microbiome heterogeneity in iCCA compared to HCC and healthy controls. Two *Veillonella* species were found to be more abundant in iCCA samples and could distinguish iCCA from HCC and healthy controls. Conversely, *Ruminococcus gnavus* was depleted in iCCA patients and could distinguish HCC from iCCA samples. High *Veillonella* genus counts in the iCCA group were associated with enriched amino acid biosynthesis and glycolysis pathways, while enriched phospholipid and thiamine metabolism pathways characterized the HCC group with high *Blautia* genus counts. These findings reveal distinct landscapes of gut dysbiosis among Thai iCCA and HCC patients and warrant further investigation as potential biomarkers.

## Introduction

Intrahepatic cholangiocarcinoma (iCCA) and hepatocellular carcinoma (HCC) are the two main histological forms of primary liver cancer (PLC). They are among the leading causes of cancer-related deaths worldwide and the most prevalent form of cancer in Thailand to date^1^. Both diseases are associated with a poor prognosis, and patients often present at an advanced, non-resectable stage. The risk factors for Thai iCCA patients include liver fluke (*Opisthorchis viverrini* – OV) infection, biliary tract disorders, and hepatitis B virus (HBV) or hepatitis C virus (HCV) infection^2^. A recent survey showed that environmental factors, combined with certain genetic polymorphisms, could also increase the risk of iCCA^3^. Risk factors for HCC in Thai patients include HBV and HCV infection^4,5^, alcohol consumption^6^, cirrhosis from any cause^4,6^, dietary aflatoxin B_1_ and other environmental exposures, with HBV alone accounting for 49% of cases^4^. To improve our understanding of disease susceptibility, progression, and patient outcomes among PLC in the Thai population, the Thailand Initiative in Genomics and Expression Research for Liver Cancer (TIGER-LC) Consortium was established^7^.

Using stored biospecimens from the TIGER-LC cohort, we previously identified several prognostic biomarkers specific to the Thai population that defined the molecular subtypes of iCCA and HCC, which suggested the possible involvement of the gut microbiome^7,8^. Given that there is a connection between the liver and intestine via the portal vein, and that individuals with chronic liver disease experience gut bacterial translocation to the liver^9^, gut microbes could be involved in the pathogenesis and progression of cancer in the liver. Previous studies on the microbiome of Thai PLC patients have focused on CCA with OV or *Helicobacter pylori* infection^10^ using tissue^11^ and bile fluid samples^12^. The gut microbiome has an advantage over tumor tissue and bile fluid microbiome because fecal matter yields higher microbial DNA biomass; hence, it is less susceptible to false positives from exogenous DNA contamination^13^. Several studies in Chinese patients have compared the gut microbiomes of iCCA and HCC directly^14–17^. However, most gut microbiome studies in iCCA and HCC to date have performed microbiome profiling using 16S rRNA genes (or amplicon) sequencing with different variable regions, which have biases towards certain taxa^18^ and often cannot accurately identify bacteria at the species level^19^. Therefore, we aimed to comprehensively characterize the gut microbiome of Thai PLC patients and healthy controls matched by age, sex, and region. To do so, we performed whole-genome metagenomic shotgun (WGMS) sequencing and identified different patterns of dysbiosis in the gut microbiome of iCCA and HCC patients in a Thai population.

## Results

### The landscape of gut dysbiosis in Thai PLC patients and healthy individuals

**Figure 1:**
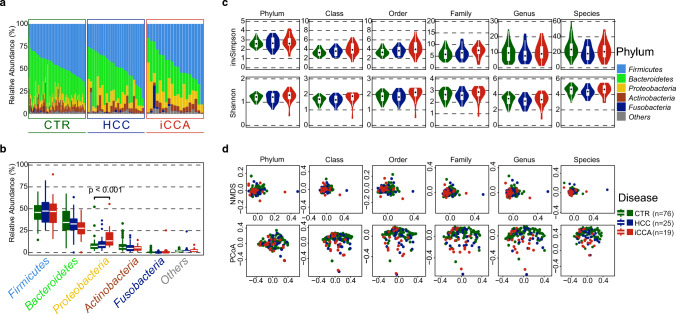
Gut dysbiosis between Thai iCCA and HCC patients have different patterns. (**a**) Relative abundance of top five phyla in stool samples from healthy controls, HCC and iCCA groups. (**b**) Relative abundance of top five phyla comparing among groups of subjects. (**c**) Alpha diversity measures among groups of subjects. (**d**) Beta diversity measures among groups of subjects based on Bray–Curtis distance metric.

The demographic and clinical characteristics of the iCCA and HCC groups were generally matched, including age, sex, BMI, and common cancer risk factors (Supplementary Table S1). There were no discernible differences in lifestyle factors known to affect the gut microbiome, such as antibiotic and antifungal use, among the three groups of subjects. We found that the gut microbiome profiles of patients with iCCA, patients with HCC, and healthy individuals were similar at the phylum level. Specifically, the phylum *Firmicutes* was the most abundant bacteria overall, followed by *Bacteroides*, *Proteobacteria, Actinobacteria,* and *Fusobacteria* (Fig. 1a). However, the relative abundance of *Proteobacteria* in iCCA patients was significantly higher than that in healthy individuals (Fig. 1b), with a similar trend in HCC patients. A full list of comparisons between the disease groups of these phyla is shown in Supplementary Table S2. Although alpha diversity measures between the three groups of subjects were not statistically different, the ranges of the Shannon–Wiener diversity index and inverse Simpson index at all taxonomic levels for iCCA were consistently larger than those for HCC (Fig. 1c), indicating a higher heterogeneity of the gut bacterial community in iCCA. In terms of beta diversity among the samples, there were no statistically significant differences between the cancer and healthy control groups, based on the Bray–Curtis distance metric, using non-negative multidimensional scaling and principal coordinates analysis (Fig. 1d). Stratified analyses based on sex and region of residence showed no bias from sex (Supplementary Fig. S1a and Table S3) or region (Supplementary Fig. S1b and Table S4). In summary, our results demonstrated that while there were no significant differences in alpha and beta diversity among iCCA, HCC, and healthy control groups, there was a trend towards higher diversity in iCCA. Additionally, at the phylum level, patients with iCCA exhibited a higher relative abundance of *Proteobacteria* than healthy individuals.

### Linear discriminant analysis identifies taxa that are specific to patient conditions

**Figure 2:**
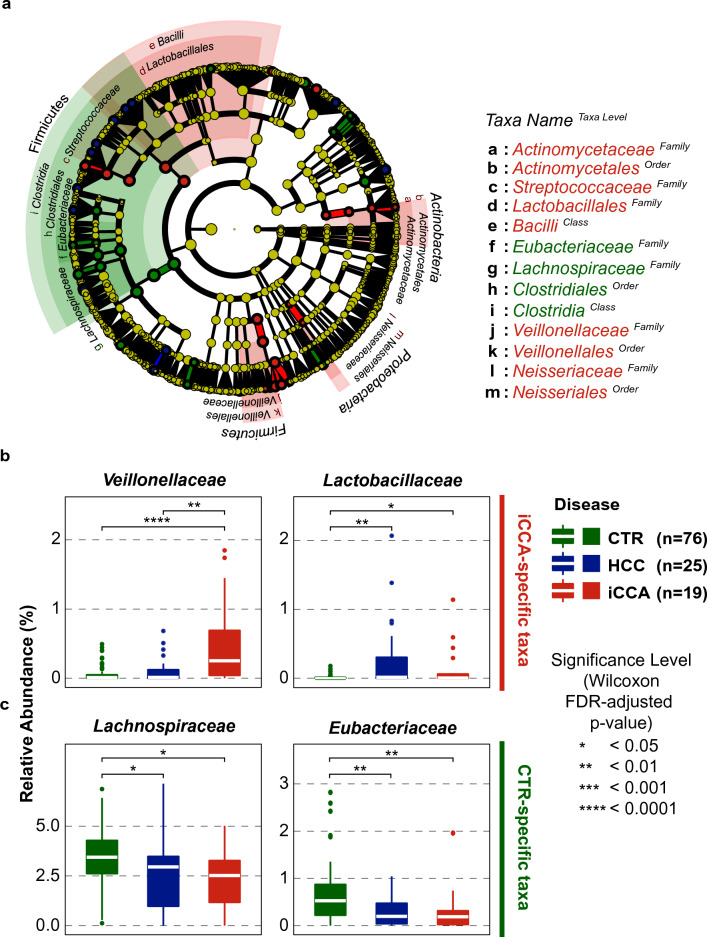
LDA identifies taxa that can differentiate disease conditions. (**a**) Overall taxa dendrogram shows the relationship and relative distance between taxa that can differentiate between groups of subjects (− log_10_[LDA score] > 2). (**b**,**c**) Four selected taxa that can differentiate iCCA (**b**) and healthy control (**c**) samples from other groups of subjects.

To identify taxa that could differentiate cancer groups from healthy controls, we utilized an alternative approach, as the phylum-level data were insufficient. We applied linear discriminant analysis (LDA) to metagenomic reads with disease condition labels using LDA Effect Size (LEfSe)^20^ and identified 61 taxa, mainly from the phyla *Firmicutes*, *Actinobacteria*, and *Proteobacteria*, which were uniquely present in each group of subjects with log_10_[LDA score] > 2 (Fig. 2a and Supplementary Table S5). The families *Veillonellaceae*, *Lactobacillales*, *Actinomycetaceae*, *Streptococcaceae*, and *Neisseriaceae* were found to differentiate iCCA samples from other groups (Fig. 2b). Meanwhile, the families *Lachnospiraceae*, *Eubacteriaceae*, and the order *Clostridiales* were able to distinguish healthy control samples from cancer groups (Fig. 2c). Notably, there were no microbe at the family level that could distinguish the HCC samples from other groups. However, the genus *Flavonifractor* has been identified as an HCC-specific taxon. The complete list of taxa identified by LEfSe is presented in Supplementary Fig. S2a and Table S5.

At the species level, the top five enriched species in the iCCA group were *Veillonella atypica, Bacteroides sp* CAG530, *Streptococcus parasanguinis, Veillonella parvula*, and *Megasphaera micronuciformis* (Supplementary Fig. S2b). The top five enriched species in the healthy control group were *Bacteroides uniformis, Anaerostipes hadrus, Blautia wexlerae, Roseburia intestinalis,* and *Phascolarctobacterium faecium* (Supplementary Fig. S2c). Finally, the top five enriched species in the HCC group were *Ruminococcus gnavus, Bifidobacterium longum, Ligilactobacillus salivarius, Streptococcus anginosus* group, and *Bacteroides finegoldii* (Supplementary Fig. S2d). These results underscore the potential of using the gut microbiome at the family level to differentiate patient groups, especially patients with iCCA, and reveal distinct patterns of species-level composition for each group of subjects.

### LDA-identified species were verified by sequence alignment and assembly-based metagenomic methods

**Figure 3:**
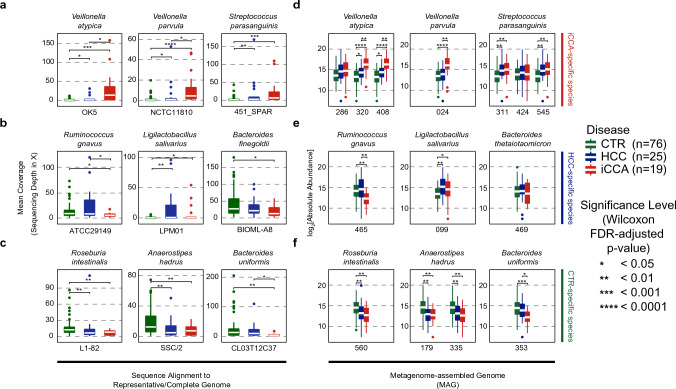
Verification of LDA-identified species by sequence alignment and assembly-based metagenomic methods. (**a**–**c**) Reads coverage (sequencing depth in X) of metagenomic reads aligned to complete and/or representative genomes of the LDA-identified species. At the top and the bottom of each boxplot is the species name and the name of complete and/or representative genome used for sequence alignment, respectively. (**d**–**f**) Log_2_[absolute abundance] of MAGs that were called and matched as iCCA-specific (**d**), HCC-specific (**d**), and healthy control-specific (**d**) species in panel (**a**–**c**). At the top and the bottom of each boxplot are the species name and MAG numbers called and matched with LDA-identified species, respectively.

To confirm the accuracy of the LDA results, additional approaches are necessary, as the marker gene-based method tends to sacrifice accuracy for speed. We employed two verification approaches for the list of species that were uniquely present in each group of subjects. First, the top three LDA-identified species from each group were selected and metagenomic reads were aligned to their complete and/or representative genomes. The presence of all nine selected species in their respective sample groups was confirmed (Fig. 3a–c). Three species specific to iCCA, namely *V. atypica*, *V. parvula*, and *S. parasanguinis*, exhibited the highest mean read coverage in iCCA samples, particularly *Veillonella species*, when compared to both HCC and healthy control samples (Fig. 3a–c). The full list of the mean sequencing coverage of all nine species is shown in Supplementary Table S6. The mean read coverage of all samples along the full genome is shown in Supplementary Fig. S3. In the second validation approach, metagenomic reads were subjected to assembly based metagenomic analysis to obtain metagenome-assembled genomes (MAGs). Fourteen MAGs were called and matched all LDA-identified species, except for *Bacteroides finegoldii* (Supplementary Table S8). Based on the absolute abundance of MAGs, five out of eight iCCA-specific MAGs were able to distinguish iCCA from the other groups (Fig. 3d and Supplementary Table S9). Notably, two MAGs, MAG320 and MAG408, matching *V. atypica* could differentiate iCCA samples from both HCC and healthy control groups. Two out of the three HCC-specific species could differentiate HCC samples from the other groups (Fig. 3e and Supplementary Table S9). All four MAGs called for control-specific species distinguished healthy control samples from other groups (Fig. 3f and Supplementary Table S9). Although the results from the two verification methods did not match perfectly at the statistical significance level, the trends and direction of change were consistent. Therefore, these results validate the presence of species identified by LDA, including disease-specific species, and their ability to differentiate diseased samples from healthy control samples.

### Pathway analysis reveals differently enriched metabolic pathways between iCCA and HCC

**Figure 4:**
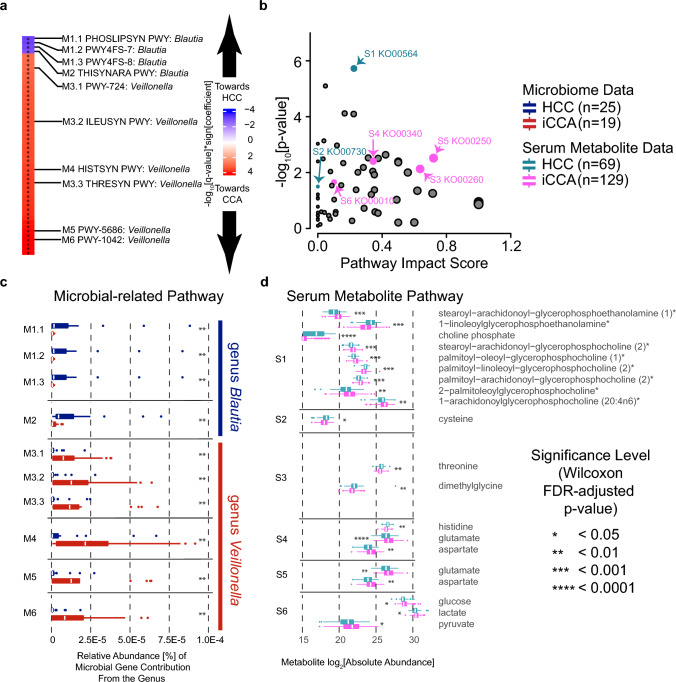
Microbial and serum metabolic pathways show different contributions between cancer groups from different genera. (**a**) Top 50 enriched microbial pathways. Pathways M1-M6 are overlapped with enriched pathways from serum metabolite pathways in panel (**b**). (**b**) Scatterplot of MSEA global test on serum metabolite data comparing iCCA and HCC patients from the TIGER-LC discovery cohort. Circle size reflects pathway impact score. Pathways S1-S6 are overlapped with enriched pathways from metagenomics data in panel (**a**). The full pathway names are listed in Supplementary Table S10. (**c**) Relative abundance of gene contribution from genus associated with the enriched microbial pathways in panel a. Pathways M1 and M2 are from genus *Blautia*, while pathways M3-M6 are from genus *Veillonella*. (**d**) Log_2_[absolute abundance] of serum metabolites from enriched metabolic pathways in panel (**b**). The metabolites shown in the figures are statistically different metabolites based on FDR-adjusted p-values.

Functional analysis of iCCA and HCC samples was conducted using microbial read and serum metabolite data. The genus *Blautia* was found to be associated with four microbial pathways (M1-M2) enriched in HCC, whereas six microbial pathways (M3-M6) associated with the genus *Veillonella* were enriched in iCCA (Fig. 4a). Six serum metabolic pathways overlapped with microbial pathways, with two pathways (S1-S2) associated with HCC, and four pathways (S3-S6) associated with iCCA (Fig. 4b). Phosphoglycerolipid metabolism (M1.1-M1.3 and S1) and thiamine metabolism pathways (M2 and S2) were enriched in HCC, while amino acid metabolism (M3.1-M3.3 and S3, and M4 and S4), nucleotide metabolism (M5 and S5), and glycolysis pathways (M6 and S6) were enriched in iCCA (Supplementary Table S10).

We also investigated differences in the contribution of microbial genes in the HCC and iCCA groups. As shown in Fig. 4c, the relative abundance of microbial gene contribution from the genus *Blautia* was statistically higher in HCC, while *Veillonella* displayed a higher relative abundance in the iCCA group. Furthermore, we observed similar trends in the absolute abundance of serum metabolites in the serum metabolic pathways, albeit to a lesser degree. Specifically, the glycerophospholipid metabolism (S1) pathway had an equal number of metabolites that were statistically different between HCC and iCCA, whereas the thiamine metabolism (S2) pathway had only one metabolite (cysteine), which was statistically higher in HCC (Fig. 4d). In contrast, pathway S3 had metabolites that were only statistically higher in the HCC group, whereas most of the metabolites in pathways S4, S5, and S6 were statistically higher in the iCCA group (Fig. 4d and Supplementary Table S10). Furthermore, analysis of covariance (ANCOVA) using disease status and risk factors as covariates to predict the metabolite outcomes showed that none of the risk factors listed in Table S1 were found to be confounding factor for any metabolite (Supplementary Table S11). Taken together, these results suggest that the gut microbiome may have metabolic consequences in patients, and our findings provide further insights into the differences between iCCA and HCC.

## Discussion

Previous studies examining the gut microbiome in Chinese cohorts with iCCA, HCC, high-risk and healthy control subjects have demonstrated significant differences in alpha and beta diversity between groups^14–17^. While alpha diversity measures and relative abundance of the main phyla of the gut microbiome did not show statistical differences between early HCC and control group^21^, HBV-related HCC and control group^22,23^, and primary CCA and control group^24^, the relative abundance of the gut microbiome at the phylum level, particularly phyla *Firmicutes* and *Bacteroidetes*, have been shown to differ between diseased and control groups in studies using 16S rRNA sequencing, a finding that was not observed in our data.

Several technical issues may have contributed to the discrepancies in the gut microbiome studies. Utilization of 16S rRNA sequencing and sequencing of different variable regions can yield different taxa recovery and accuracy rates of taxa identification^25^, resulting in conflicting evidence. Additionally, differences in the databases used for calling the taxa can affect the accuracy of taxon recovery from 16S rRNA sequencing^26^. In contrast, WGMS sequencing may be more accurate in terms of the number of recovered taxa and accuracy^19,27^. A previous study found that different sequencing methods alone can explain the discrepancy in the alpha diversity of the human infant gut microbiome^18^. Furthermore, the gut microbiome is known to be influenced by diet^28^, as has been observed in the Thai population^29,30^. Although differences in etiology between geographical areas can lead to inconsistencies in studies^31^, we did not observe differences between regions within Thailand, which are associated with different food intake patterns^30^. Thus, further assessment of the gut microbiome of healthy cohorts from various regions in Thailand using WGMS will provide a clearer understanding of the relationship between diet and the gut microbiome.

*Veillonella* sp. has been identified in the gut microbiome of patients with biliary tract diseases such as primary sclerosing cholangitis (PSC)^32^, biliary atresia^33^, and liver fluke infection^34^. In our study, we also observed increased *Veillonella* sp., specifically *V. atypica* and *V. parvula,* in the gut of patients with iCCA. Similarly, a study on nonalcoholic steatohepatitis (NASH) patients identified the *Veillonella* genus as a biomarker for treatment response to a hormone analog^35^. Additionally, a higher relative abundance of the family *Veillonellaceae* was found in a cohort of Chinese patients with CCA, and the genus *Veillonella* is one of an eight-genera predictive signature that can differentiate CCA from HCC and healthy control groups^15^. Taken together, the results from these studies suggest that increased *Veillonella* sp. in the gut, particularly *V. atypica,* is associated with biliary tract diseases and could potentially serve as a fecal biomarker for CCA. Notably, increased levels of the genera *Veillonella* and *Streptococcus* were reported in the saliva of CCA patients compared to healthy controls^36^, suggesting that the species found in the gut in our study might have migrated from the mouth to the gut of the patients themselves, although the underlying mechanism remains unknown.

Very few studies have performed microbial gene contribution and functional analysis on microbiome data. The reason might be that most of these studies used 16S rRNA sequencing, and the conclusions or interpretations that can be drawn from pathway analysis results based on 16S rRNA data are limited. The enriched microbial pathways found in our study are known to be altered in various diseases, including cancer. For example, several serum metabolites from the glycerophospholipid metabolism (S1) pathway were identified as HCC-specific metabolites in our previous study, that is, stearoyl-linoleoyl-glycerophosphocholine, palmitoyl-linoleoyl-glycerophosphocholine, 2-palmitoylglycerophosphocholine, and 1-arachidonoylglycerophosphocholine^8^. The enrichment of *Veillonella* sp. in the gut is correlated with enhanced marathon running performance via *V. atypica*’s capacity to metabolize serum lactate that entered the gut lumen, resulting in the generation of acetate and propionate^37^. However, we can only infer an association between serum and microbiome data, as they were obtained from different patients. While some studies have profiled the microbiome and metabolites together in the same fecal samples, the class of metabolites measured has been limited to one class of metabolites^14^. Therefore, comprehensive metabolomic profiling directly from fecal samples together with microbiome profiling of the same patients is needed to better understand the association between the microbiome and metabolic consequences in cancer.

The scope of our study was limited by the absence of samples from intermediate- or high-risk patient groups such as those with OV infection and primary sclerosing cholangitis (PSC) for iCCA or HBV/HCV infection, liver cirrhosis, chronic liver disease (CLD), nonalcoholic steatohepatitis (NASH), and nonalcoholic fatty liver disease (NAFLD) for HCC. Studying microbiomes of the high-risk groups might help us discern whether certain species are indeed associated with primary liver cancer. In addition, a direct comparison of our results with those of other studies might not be possible or meaningful due to differences in sequencing methods. Furthermore, the OV infection status of our samples was inferred from a questionnaire rather than a definitive test, such as ova and parasite stool examination. Although the diagnosis at the time of recruitment of iCCA patients suggested intrahepatic origin of the CCA tissues, we cannot definitively exclude the presence of OV infection in the fecal samples. Since the OV-PCR test requires a substantial amount of fecal material and modified DNA extraction protocols to obtain OV-DNA by breaking the OV-egg, the thin smear stool collection method employed in this study using an FOBT card lacks sufficient material for the OV-PCR test. Finally, the number of samples from the cancer groups was limited, and further analyses with a larger sample size are needed.

In conclusion, our study indicates that gut dysbiosis landscapes and disease-specific fecal microbial species differ between iCCA and HCC patients. As such, these microbial species may hold potential as noninvasive biomarkers for the early detection of primary liver cancer.

## Materials and methods

### Patient recruitment and specimen collection

Stool samples from 120 patients were collected from 4 clinical centers in northern, northeastern, and central Thailand. The cohort consisted of 19 iCCA, 25 HCC, and 76 healthy individuals that were matched by age, sex, and region of residence to cancer cases. Clinical, socioeconomic, and demographic data were extracted from the comprehensive questionnaires and medical records collected at the time of recruitment. Patients with iCCA were diagnosed using a combination of imaging and histological investigations. HCC patients were diagnosed using combinations of imaging studies, tumor size, alpha-fetoprotein (AFP) levels, and histological investigations. Healthy controls were individuals without a history of cancer who were mostly recruited during regular physical checkups and other routine procedures. Informed consent was obtained from all patients included in this study, and the protocols were approved by the Institutional Review Boards of the respective institutions (NCI protocol number 13CN089; CRI protocol number 18/2555; Chulabhorn Hospital protocol number 11/2553; Thai NCI protocol number EC163/2010; Chiang Mai University protocol number TIGER-LC; Khon Kaen University protocol number HE541099). Fecal samples were collected prior to any treatment on a Hema-Screen Occult Blood Rapid Test card (Stanbio Laboratory, Boerne, TX, USA), according to the manufacturer’s instructions. The cards were kept at − 80 °C without further processing.

### DNA extraction and WGMS sequencing

One square of fecal occult blood test card containing a thin smear of stool sample was placed into bead-beating tubes and microbial DNA was extracted using the ZymoBIOMICS DNA Miniprep Kit (Zymo Research, Irvine, CA, USA) according to the manufacturer’s protocol. Sequencing library construction of microbial DNA was prepared by random fragmentation followed by 5’ and 3’ adapter ligation using the Nextera XT DNA Library Preparation Kit (Illumina, San Diego, CA, USA). WGMS was performed on one lane of the flow cell using the NovaSeq platform (Illumina) at a read length of 150 base pairs (bp) in paired-end mode. The average yield per sample was approximately 40 million reads.

### Data pre-processing

MultiQC^38^ was used for sequence quality checks, and Trimmomatic^39^ was used for sequencing adapter trimming steps. The average number of reads that passed Q30 was approximately 39 million per sample. The host-genome removal step was performed by aligning the reads to the human genome version GRCh38^40^ using Bowtie2^41^, which yielded an average of 18 million reads per sample and was then used for all downstream analyses. The data were deposited in the NCBI SRA with accession number PRJNA932948.

### Marker gene-based metagenomic analysis

Centrifuge^42^ and MetaPhlAn3^43^ were used to perform marker gene-based metagenomic analyses, both with default settings. Centrifuge was run with the h + p + v + c database derived from the NCBI nucleotide (nt) database, which includes human, prokaryote, viral, and 106 complete SARS-CoV-2 genomes [database dated March 29, 2020]. Approximately 50% of the reads were classified as microbial. MetaPhlAn3 was run using the ChocoPhlAn database^43^ version 201901b. Linear discriminant analysis (LDA) was performed using LDA Effect Size (LEfSe) with default settings^20^ to identify taxa that could differentiate samples into groups. The top three species in each group were selected for verification.

### Sequence alignment to complete and/or representative genomes

The complete and/or representative genomes of the top three species selected by LDA for each disease condition were retrieved from the NCBI database. Priority was given to complete genome classification over representative genome designation. If no complete genome was available, the representative genome was used. The complete and representative genomes used in this study are listed in Supplementary Table S7. All representative or complete genome contigs were concatenated into one FASTA file, and metagenomic reads were aligned to the FASTA file. BWA-MEM2^44^ was used for the sequence alignment. The results from sequence alignment were visualized, and the mean sequencing depth of each species in each disease condition was calculated by Anvi’o^45^.

### Assembly-based metagenomic analysis

Assembly based metagenomic analysis was performed using ATLAS^46^, a collection of tools based on the Snakemake pipeline language^47^. The pipeline was run sequentially on all samples to generate a single combined metagenome-assembled genome (MAG) library that contained all species present in the samples. Boxplots of absolute abundance of MAGs that matched or were closely related to the selected species from LDA were generated using *ggplot2* package in R^48^.

### Microbial metabolic pathway analysis

Data from MetaPhlAn3 were further used for functional potential profiling of microbial communities using HUMAnN3^43^ based on the UniRef gene family database^49^ and the MetaCyc Metabolic Pathway Database^50^. The associations between the sample metadata and functional potential data were determined using MaAsLin2^51^. The HCC group was used as a reference. P-values of all associations were adjusted for multiple hypothesis testing using false discovery rate (FDR) correction and the Holm-Bonferroni procedure, and the top 50 enriched microbial metabolic pathways were selected.

Serum metabolite data from the TIGER-LC discovery cohort in our previous studies^7,8^ were used to perform metabolite set enrichment analysis (MSEA) between iCCA and HCC groups using Global test, an empirical Bayesian generalized linear model^52^, in MetaboAnalyst 5.0 platform^53^ with the KEGG pathway database^54^. The HCC group was used as the reference group. The enriched microbial (M) and serum (S) metabolic pathways involved in the same processes were deemed to be overlapping pathways between microbial and serum metabolites.

### Statistical analysis

All statistical analyses were performed using R version 4.0^55^. The Shannon–Wiener diversity index, inverse Simpson index (within-sample or alpha diversity), and Bray–Curtis distance (between-sample or beta diversity) were calculated using the *Vegan* package^56^. Stacked bar plots, box plots, non-metric multidimensional scaling (NMDS), and principal coordinate analysis (PCoA) plots were generated using *ggplot2*^48^. All reported p-values were two-sided p-values calculated by Wilcoxon rank-sum test (Mann–Whitney *U* test) between groups, using *rstatix* package^57^ with FDR correction using the Holm-Bonferroni procedure^58^. Confounding factor correction for serum metabolomics data was calculated by ANCOVA.

### Statement of ethics

Written informed consent was obtained from all patients included in this study in accordance with the Declaration of Helsinki and Good Clinical Practice guidelines. The study protocols were approved by the Institutional Review Boards of the respective institutions (NCI protocol number 13CN089; CRI protocol number 18/2555; Chulabhorn Hospital protocol number 11/2553; Thai NCI protocol number EC163/2010; Chiang Mai University protocol number TIGER-LC; Khon Kaen University protocol number HE541099).

## Supplementary Information

Supplementary Information.

## Data Availability

All data needed to evaluate the conclusions in the paper are presented in the paper and/or Supplementary Materials. Additional data related to this study are available upon reasonable request from the corresponding author. Raw metagenomic sequences can be downloaded from SRA database with accession number PRJNA932948.
