# Supplementary material for: Gut dysbiosis in Thai intrahepatic cholangiocarcinoma and hepatocellular carcinoma
Source: Sci Rep. 2023 Jul 14;13:11406. doi: 10.1038/s41598-023-38307-2 (PMC10349051; doi:10.1038/s41598-023-38307-2)
Supplement: Supplementary file 1 — Supplementary Information. [file 41598_2023_38307_MOESM1_ESM.pdf]

Supplementary Information

Gut dysbiosis in Thai intrahepatic cholangiocarcinoma and hepatocellular carcinoma

Yotsawat Pomyen[1], Jittiporn Chaisaingmongkol[2,3], Siritida Rabibhadana[2], Benjarath Pupacdi[1], Donlaporn Sripan[2], Chidchanok Chornkrathok[2], Anuradha Budhu[4,5], Vajarabhongsa Budhisawasdi[2,6], Nirush Lertprasertsuke[7], Anon Chotirosniramit[7], Chawalit Pairojkul[6], Chirayu U. Auewarakul[8], Teerapat Ungtrakul[8], Thaniya Sricharunrat[9], Kannikar Phornphutkul[10], Suleeporn Sangrajang[11], Christopher A. Loffredo[12], Curtis C. Harris[5], Chulabhorn Mahidol[2], Xin Wei Wang[4,5], Mathuros Ruchirawat[2,3] & TIGER-LC Consortium

This file includes:

Supplementary Figures: Figs. S1 to S3
Supplementary Tables: Tables S1 to S11

Figure S1 Relative abundance of top 5 phyla stratified by sex and region

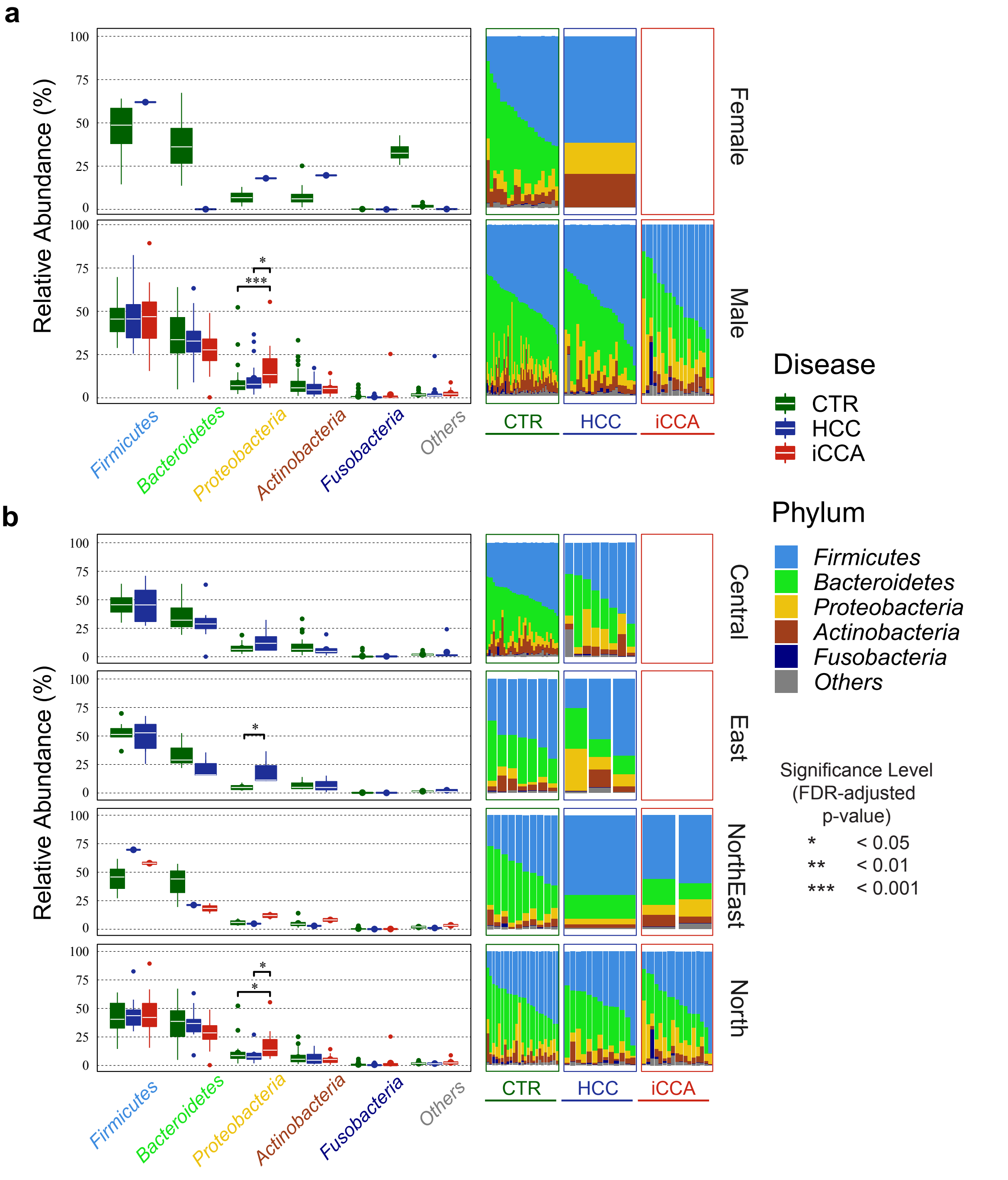

Fig. S1. Relative abundance of top five phyla stratified by sex and region.

(**a-b**) Relative abundance of the five phyla, stratified by disease condition and sex (**a**), and disease and region (**b**). There is no discernible difference between overall pattern after stratification by sex and region of the resident of the patients. Abbreviations: CTR – healthy control; HCC – hepatocellular carcinoma; iCCA – intrahepatic cholangiocarcinoma; NE – Northeast.

Figure S2 LDA results and feature plots from LEfSe

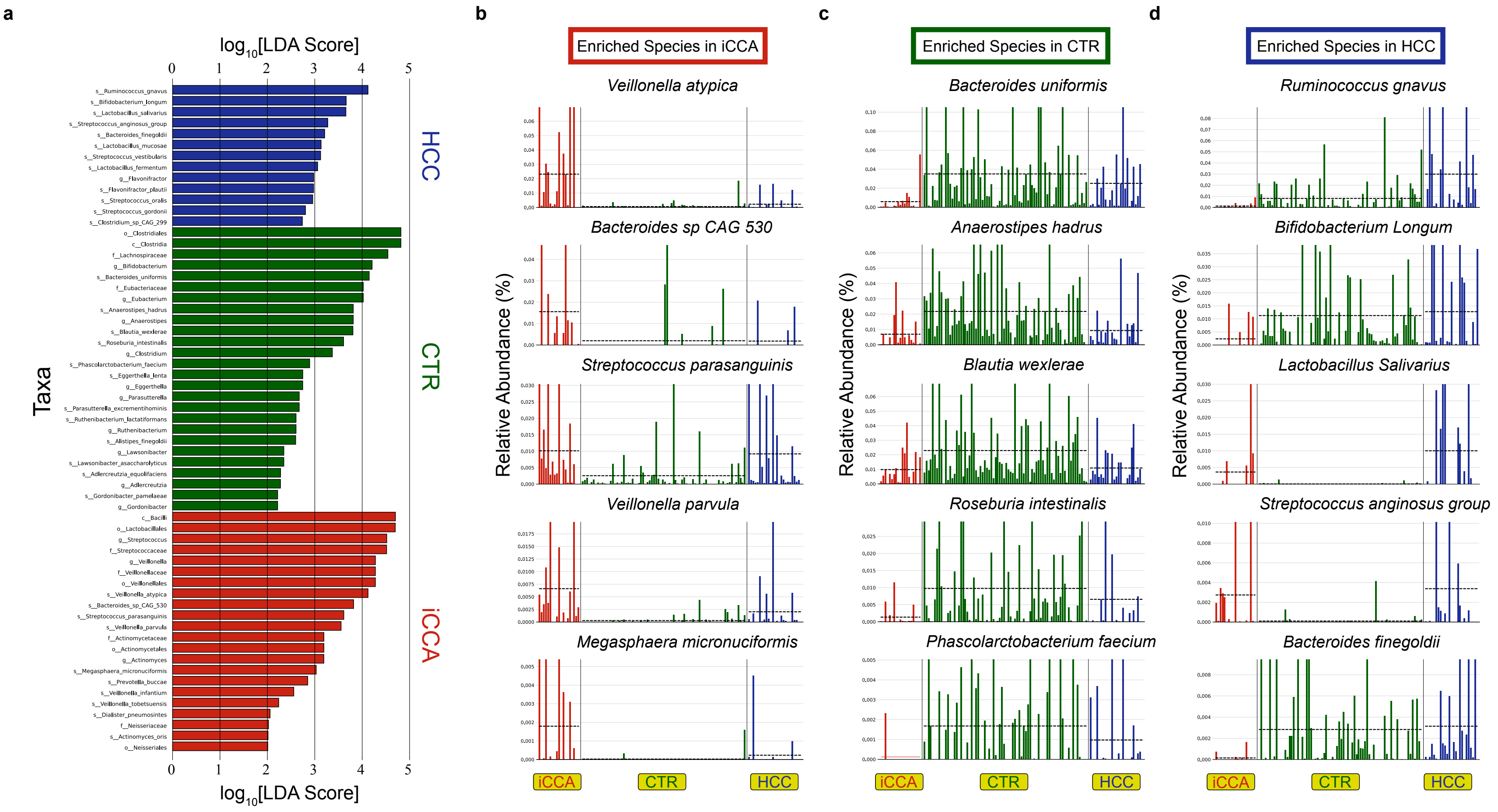

Fig. S2. LDA results and feature plots from LEfSe.

(**a**) Barplots of $\log_2$[LDA score] of disease-specific taxa identified by LEfSe. (**b-d**) Feature plots of top five species based on LDA scores that are specific to iCCA (**b**), healthy control (**c**), and HCC (**d**) groups.

Figure S3 Mean read coverage from sequence alignment in all samples

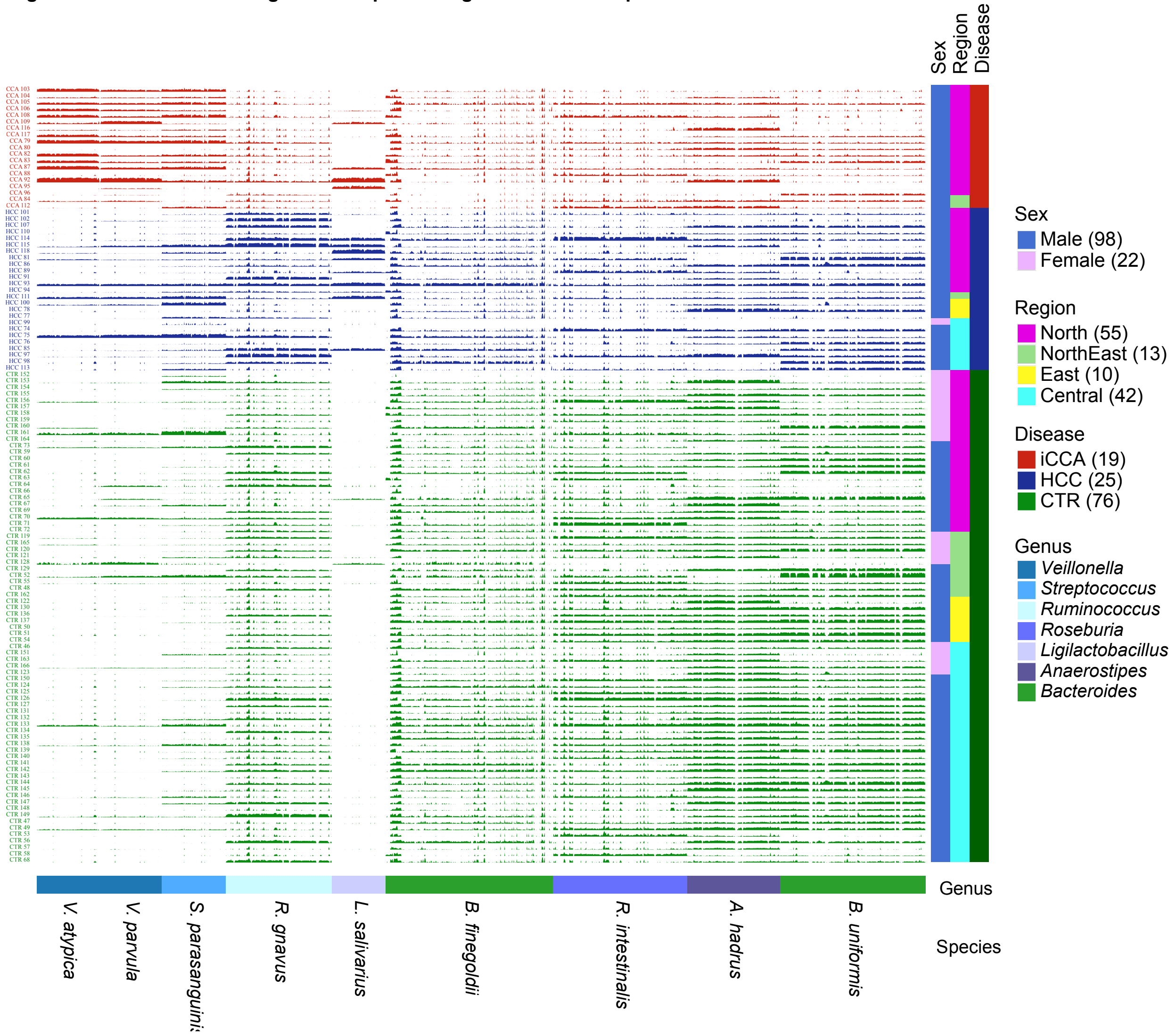

Fig. S3. Mean read coverage from sequence alignment in all 120 samples.

The right annotation columns are sex, regions of residency, and disease condition of the samples. The bottom annotation rows are genus and species names.

Supplementary Tables

Table S1. Demographic, clinical and laboratory characteristics of iCCA, HCC patients and healthy controls in the TIGER-LC cohort at the time of surgery.

Clinical variable	iCCA	HCC	CTR	*p*-value**	
	(n = 19)	(n = 25)	(n=76)	iCCA vs HCC	all groups
**Demographic**					
**Sex**				1	<0.01
Male	19	24	21		
Female	0	1	55		
**Mean Age (range)**	59 (40-74)	57 (39-73)	57 (39-74)	0.45	0.44
≥50 yr	17	19	58		
<50 yr	2	6	18		
**Regions**				<0.01	<0.01
North	17	13	25		
Northeast	2	1	10		
Central	0	8	34		
East	0	3	7		
**Risk factors**					
**Body Mass Index**				0.22	<0.01
Under weight, BMI <18.5	5	2	1		
Normal weight, BMI = 18.5–23.9	10	18	28		
Overweight/Obesity, BMI >24	3	5	47		
**Agriculture jobs**				0.84	<0.01
Yes	15	19	21		
No	3	6	55		
**Smoking status**				1	<0.01
Never	4	5	42		
Ever	14	20	34		
**Currently Smoking**				1	0.51
No	15	22	70		
Yes	3	3	6		
**Alcohol consumption**				0.33	0.06
No	2	0	14		
Yes	16	25	62		
**HBV status**				<0.01	<0.01
Nonviral	6	8	49		
Chronic Carrier	11	3	26		
Active viral replication chronic carrier	1	14	1		
**HCV status**				0.34	0.02
Negative	18	21	75		
Positive	0	3	1		
**History of *Opisthorchis viverrini***				0.7	0.29
No	13	18	67		
Yes	0	1	1		
Unknown	4	6	8		

Table S1. (continued)

Clinical variable	iCCA	HCC	CTR	*p*-value**	
	(n = 19)	(n = 25)	n=76	iCCA vs HCC	all groups
**Family history of cancer**				0.06	0.06
No	11	8	44		
Yes	5	16	30		
Not sure	2	1	2		
*noone has history of cancer					
**History of diabetes**				0.08	0.02
No	17	18	71		
Yes	0	6	4		
Unknown	1	1	1		
*noone has history of insulin use					
**Antibiotic use**				1	0.12
No	12	15	31		
Yes	6	9	39		
**Antifungal use**				1	0.68
No	18	21	68		
Yes	0	1	2		
**Antiviral use**				0.01	<0.01
No	18	15	69		
Yes	0	9	1		
**Chonic dental diesease**				0.58	0.57
No	16	20	61		
Yes	1	4	8		
**Gastrointestinal disease**				1	0.75
No	10	16	58		
Yes	0	1	3		
**Liver function factors**					
**Child-Pugh class**				<0.01	<0.01
A	2	17	0		
B	15	5	0		
C	1	0	0		
**Insulin treated**				1	0.15
Yes	0	1	0		
No	19	24	76		
**Fasting blood sugar status**				1	0.4
Low, < 70mg/dL	0	0	3		
Normal, 70-110mg/dL	12	16	58		
High, > 110mg/dL	6	8	15		
**INR (International normalized ratio)**				0.16	<0.01
Normal, <1.1	11	8	56		
Abnormal, ≥1.1	8	17	2		
**Total bilirubin**				<0.01	<0.01
Normal, ≤1.9mg/dL	4	24	76		
Abnormal, ≥1.9mg/dL	15	1	0		

Table S1. (continued)

Clinical variable	iCCA	HCC	CTR	*p*-value**	
	(n = 19)	(n = 25)	(n=76)	iCCA vs HCC	all groups
**ALT (Alanine aminotransferase)**				0.87	<0.01
Normal, <50U/L	7	11	74		
Abnormal, ≥50U/L	12	14	2		
**ALP (Alkaline phosphatase)**				<0.01	<0.01
Normal, < 147U/L	2	15	76		
Abnormal, ≥147U	17	10	0		
**CA19-9**				0.58	<0.01
Normal, < 37U/mL	7	12	58		
Abnormal, ≥37U	12	12	0		
**AFP (Alpha-fetoprotein)**				<0.01	<0.01
Normal, <300 ng/mL	18	12	76		
Abnormal, ≥300 ng	0	13	0		

Abbreviations
iCCA: intrahepatic cholangiocarcinoma
HCC: hepatocellular carcinoma
CTR: healthy control

****P values indicate the significance of difference among groups**
Pearson's chi-squared test was used to compare categorical variables.

Table S2. FDR-adjusted p-values from Wilcoxon Rank Sum test between groups of subjects for top five phyla and the remaining phyla.

Phylum	Group 1	Group 2	FDR-adjusted p-value	Significance level
**Firmicutes**	CTR	HCC	0.997	ns
	CTR	iCCA	0.997	ns
	HCC	iCCA	0.997	ns
**Bacteroidetes**	CTR	HCC	0.304	ns
	CTR	iCCA	0.067	ns
	HCC	iCCA	0.304	ns
**Proteobacteria**	CTR	HCC	0.055	ns
	CTR	iCCA	**0.000152**	***
	HCC	iCCA	0.055	ns
**Actinobacteria**	CTR	HCC	0.332	ns
	CTR	iCCA	0.332	ns
	HCC	iCCA	0.815	ns
**Fusobacteria**	CTR	HCC	0.584	ns
	CTR	iCCA	0.579	ns
	HCC	iCCA	0.69	ns
**Others**	CTR	HCC	0.228	ns
	CTR	iCCA	0.228	ns
	HCC	iCCA	0.228	ns

Abbreviations
N/A: not applicable
ns: not significant
FDR: false discovery rate
HCC: hepatocellular carcinoma
iCCA: intrahepatic cholangiocarcinoma
CTR: healthy control

Table S3. FDR-adjusted p-values from Wilcoxon Rank Sum test between groups of subjects for top five phyla and the remaining phyla, stratified by sex.

sex	Phylum	Group1	Group2	FDR-adjusted p-value	Significance Level
Female	Firmicutes	CTR	HCC	0.273	ns
		CTR	iCCA	N/A	N/A
		HCC	iCCA	N/A	N/A
	Bacteroidetes	CTR	HCC	0.091	ns
		CTR	iCCA	N/A	N/A
		HCC	iCCA	N/A	N/A
	Proteobacteria	CTR	HCC	0.091	ns
		CTR	iCCA	N/A	N/A
		HCC	iCCA	N/A	N/A
	Actinobacteria	CTR	HCC	0.182	ns
		CTR	iCCA	N/A	N/A
		HCC	iCCA	N/A	N/A
	Fusobacteria	CTR	HCC	0.091	ns
		CTR	iCCA	N/A	N/A
		HCC	iCCA	N/A	N/A
	Others	CTR	HCC	0.091	ns
		CTR	iCCA	N/A	N/A
		HCC	iCCA	N/A	N/A
Male	Firmicutes	CTR	HCC	0.912	ns
		CTR	iCCA	0.912	ns
		HCC	iCCA	0.912	ns
	Bacteroidetes	CTR	HCC	0.568	ns
		CTR	iCCA	0.116	ns
		HCC	iCCA	0.277	ns
	Proteobacteria	CTR	HCC	0.116	ns
		CTR	iCCA	**0.000339**	***
		HCC	iCCA	**0.044**	*
	Actinobacteria	CTR	HCC	0.241	ns
		CTR	iCCA	0.378	ns
		HCC	iCCA	0.637	ns
	Fusobacteria	CTR	HCC	0.728	ns
		CTR	iCCA	0.728	ns
		HCC	iCCA	0.837	ns
	Others	CTR	HCC	0.324	ns
		CTR	iCCA	0.324	ns
		HCC	iCCA	0.324	ns

Table S4. FDR-adjusted p-values from Wilcoxon Rank Sum test between groups of subjects for top five phyla and the remaining phyla, stratified by region.

Region	Phylum	Group1	Group2	FDR-adjusted p-value	Significance Level
**Central**	Firmicutes	CTR	HCC	0.888	ns
		CTR	iCCA	N/A	N/A
		HCC	iCCA	N/A	N/A
	Bacteroidetes	CTR	HCC	0.368	ns
		CTR	iCCA	N/A	N/A
		HCC	iCCA	N/A	N/A
	Proteobacteria	CTR	HCC	0.1	ns
		CTR	iCCA	N/A	N/A
		HCC	iCCA	N/A	N/A
	Actinobacteria	CTR	HCC	0.199	ns
		CTR	iCCA	N/A	N/A
		HCC	iCCA	N/A	N/A
	Fusobacteria	CTR	HCC	0.863	ns
		CTR	iCCA	N/A	N/A
		HCC	iCCA	N/A	N/A
	Others	CTR	HCC	0.21	ns
		CTR	iCCA	N/A	N/A
		HCC	iCCA	N/A	N/A
**East**	Firmicutes	CTR	HCC	1	ns
		CTR	iCCA	N/A	N/A
		HCC	iCCA	N/A	N/A
	Bacteroidetes	CTR	HCC	0.183	ns
		CTR	iCCA	N/A	N/A
		HCC	iCCA	N/A	N/A
	Proteobacteria	CTR	HCC	**0.017**	*
		CTR	iCCA	N/A	N/A
		HCC	iCCA	N/A	N/A
	Actinobacteria	CTR	HCC	1	ns
		CTR	iCCA	N/A	N/A
		HCC	iCCA	N/A	N/A
	Fusobacteria	CTR	HCC	1	ns
		CTR	iCCA	N/A	N/A
		HCC	iCCA	N/A	N/A
	Others	CTR	HCC	1	ns
		CTR	iCCA	N/A	N/A
		HCC	iCCA	N/A	N/A

Table S4. (continued)

Region	Phylum	Group1	Group2	FDR-adjusted p-value	Significance Level
**Northeast**	Firmicutes	CTR	HCC	0.41	ns
		CTR	iCCA	0.41	ns
		HCC	iCCA	0.667	ns
	Bacteroidetes	CTR	HCC	0.546	ns
		CTR	iCCA	0.182	ns
		HCC	iCCA	1	ns
	Proteobacteria	CTR	HCC	0.909	ns
		CTR	iCCA	0.363	ns
		HCC	iCCA	0.909	ns
	Actinobacteria	CTR	HCC	0.667	ns
		CTR	iCCA	0.667	ns
		HCC	iCCA	0.667	ns
	Fusobacteria	CTR	HCC	1	ns
		CTR	iCCA	1	ns
		HCC	iCCA	1	ns
	Others	CTR	HCC	0.727	ns
		CTR	iCCA	0.727	ns
		HCC	iCCA	0.727	ns
**North**	Firmicutes	CTR	HCC	0.98	ns
		CTR	iCCA	0.98	ns
		HCC	iCCA	0.98	ns
	Bacteroidetes	CTR	HCC	0.903	ns
		CTR	iCCA	0.178	ns
		HCC	iCCA	0.178	ns
	Proteobacteria	CTR	HCC	0.605	ns
		CTR	iCCA	**0.042**	*
		HCC	iCCA	**0.042**	*
	Actinobacteria	CTR	HCC	0.734	ns
		CTR	iCCA	0.734	ns
		HCC	iCCA	0.967	ns
	Fusobacteria	CTR	HCC	0.869	ns
		CTR	iCCA	0.869	ns
		HCC	iCCA	0.869	ns
	others	CTR	HCC	0.564	ns
		CTR	iCCA	0.564	ns
		HCC	iCCA	0.564	ns

Table S5. LDA scores from LEfSe.

Taxon	Disease	LDA Score	p-val
k__Bacteria.p__Firmicutes.c__Bacilli	iCCA	4.701	1.23E-05
k__Bacteria.p__Firmicutes.c__Bacilli.o__Lactobacillales	iCCA	4.698	1.18E-05
k__Bacteria.p__Firmicutes.c__Bacilli.o__Lactobacillales.f__Streptococcaceae.g__Streptococcus	iCCA	4.518	0.00269814
k__Bacteria.p__Firmicutes.c__Bacilli.o__Lactobacillales.f__Streptococcaceae	iCCA	4.515	0.0033373
k__Bacteria.p__Firmicutes.c__Negativicutes.o__Veillonellales.f__Veillonellaceae.g__Veillonella	iCCA	4.278	6.79E-07
k__Bacteria.p__Firmicutes.c__Negativicutes.o__Veillonellales	iCCA	4.278	5.81E-05
k__Bacteria.p__Firmicutes.c__Negativicutes.o__Veillonellales.f__Veillonellaceae	iCCA	4.278	5.81E-05
k__Bacteria.p__Firmicutes.c__Negativicutes.o__Veillonellales.f__Veillonellaceae.g__Veillonella.s__Veillonella_atypica	iCCA	4.123	4.47E-06
k__Bacteria.p__Bacteroidetes.c__Bacteroidia.o__Bacteroidales.f__Bacteroidaceae.g__Bacteroides.s__Bacteroides_sp_CAG_530	iCCA	3.819	9.93E-05
k__Bacteria.p__Firmicutes.c__Bacilli.o__Lactobacillales.f__Streptococcaceae.g__Streptococcus.s__Streptococcus_parasanguinis	iCCA	3.612	0.00020069
k__Bacteria.p__Firmicutes.c__Negativicutes.o__Veillonellales.f__Veillonellaceae.g__Veillonella.s__Veillonella_parvula	iCCA	3.555	3.91E-07
k__Bacteria.p__Actinobacteria.c__Actinobacteria.o__Actinomycetales	iCCA	3.192	0.00022576
k__Bacteria.p__Actinobacteria.c__Actinobacteria.o__Actinomycetales.f__Actinomycetaceae	iCCA	3.192	0.00022576
k__Bacteria.p__Actinobacteria.c__Actinobacteria.o__Actinomycetales.f__Actinomycetaceae.g__Actinomyces	iCCA	3.191	0.00040716
k__Bacteria.p__Firmicutes.c__Negativicutes.o__Veillonellales.f__Veillonellaceae.g__Megasphaera.s__Megasphaera_micronuciformis	iCCA	3.030	2.52E-06
k__Bacteria.p__Bacteroidetes.c__Bacteroidia.o__Bacteroidales.f__Prevotellaceae.g__Prevotella.s__Prevotella_buccae	iCCA	2.851	0.00461598
k__Bacteria.p__Firmicutes.c__Negativicutes.o__Veillonellales.f__Veillonellaceae.g__Veillonella.s__Veillonella_infantium	iCCA	2.557	0.00022515
k__Bacteria.p__Firmicutes.c__Negativicutes.o__Veillonellales.f__Veillonellaceae.g__Veillonella.s__Veillonella_tobetsuensis	iCCA	2.238	1.29E-05
k__Bacteria.p__Firmicutes.c__Negativicutes.o__Veillonellales.f__Veillonellaceae.g__Dialister.s__Dialister_pneumosintes	iCCA	2.057	2.59E-09
k__Bacteria.p__Proteobacteria.c__Betaproteobacteria.o__Neisseriales.f__Neisseriaceae	iCCA	2.024	1.24E-06
k__Bacteria.p__Actinobacteria.c__Actinobacteria.o__Actinomycetales.f__Actinomycetaceae.g__Actinomyces.s__Actinomyces_oris	iCCA	2.014	0.00797876
k__Bacteria.p__Proteobacteria.c__Betaproteobacteria.o__Neisseriales	iCCA	2.008	1.24E-06

Table S5. (continued)

Taxon	Disease	LDA Score	p-val
k__Bacteria.p__Firmicutes.c__Clostridia	CTR	4.817	0.00088887
k__Bacteria.p__Firmicutes.c__Clostridia.o__Clostridiales	CTR	4.817	0.00088887
k__Bacteria.p__Firmicutes.c__Clostridia.o__Clostridiales.f__Lachnospiraceae	CTR	4.542	0.004241
k__Bacteria.p__Actinobacteria.c__Actinobacteria.o__Bifidobacteriales.f__Bifidobacteriaceae.g__Bifidobacterium	CTR	4.209	0.00475378
k__Bacteria.p__Bacteroidetes.c__Bacteroidia.o__Bacteroidales.f__Bacteroidaceae.g__Bacteroides.s__Bacteroides_uniformis	CTR	4.148	0.00021085
k__Bacteria.p__Firmicutes.c__Clostridia.o__Clostridiales.f__Eubacteriaceae	CTR	4.023	0.00038044
k__Bacteria.p__Firmicutes.c__Clostridia.o__Clostridiales.f__Eubacteriaceae.g__Eubacterium	CTR	4.023	0.00038044
k__Bacteria.p__Firmicutes.c__Clostridia.o__Clostridiales.f__Lachnospiraceae.g__Anaerostipes.s__Anaerostipes_hadrus	CTR	3.812	0.0001303
k__Bacteria.p__Firmicutes.c__Clostridia.o__Clostridiales.f__Lachnospiraceae.g__Anaerostipes	CTR	3.812	0.00010994
k__Bacteria.p__Firmicutes.c__Clostridia.o__Clostridiales.f__Lachnospiraceae.g__Blautia.s__Blautia_wexlerae	CTR	3.804	0.00126701
k__Bacteria.p__Firmicutes.c__Clostridia.o__Clostridiales.f__Lachnospiraceae.g__Roseburia.s__Roseburia_intestinalis	CTR	3.607	0.00172901
k__Bacteria.p__Firmicutes.c__Clostridia.o__Clostridiales.f__Clostridiaceae.g__Clostridium	CTR	3.371	0.00058663
k__Bacteria.p__Firmicutes.c__Negativicutes.o__Acidaminococcales.f__Acidaminococcaceae.g__Phascolarctobacterium.s__Phascolarctobacterium_faecium	CTR	2.892	0.00197922
k__Bacteria.p__Actinobacteria.c__Coriobacteriia.o__Eggerthellales.f__Eggerthellaceae.g__Eggerthella.s__Eggerthella_lenta	CTR	2.746	0.00124599
k__Bacteria.p__Actinobacteria.c__Coriobacteriia.o__Eggerthellales.f__Eggerthellaceae.g__Eggerthella	CTR	2.745	0.00124599
k__Bacteria.p__Proteobacteria.c__Betaproteobacteria.o__Burkholderiales.f__Sutterellaceae.g__Parasutterella	CTR	2.672	0.00305573
k__Bacteria.p__Proteobacteria.c__Betaproteobacteria.o__Burkholderiales.f__Sutterellaceae.g__Parasutterella.s__Parasutterella_excrementihominis	CTR	2.672	0.00305573
k__Bacteria.p__Firmicutes.c__Clostridia.o__Clostridiales.f__Ruminococcaceae.g__Ruthenibacterium.s__Ruthenibacterium_lactatiformans	CTR	2.604	2.62E-05
k__Bacteria.p__Firmicutes.c__Clostridia.o__Clostridiales.f__Ruminococcaceae.g__Ruthenibacterium	CTR	2.604	2.62E-05
k__Bacteria.p__Bacteroidetes.c__Bacteroidia.o__Bacteroidales.f__Rikenellaceae.g__Alistipes.s__Alistipes_finegoldii	CTR	2.597	0.00279253
k__Bacteria.p__Firmicutes.c__Clostridia.o__Clostridiales.f__Clostridiales_unclassified.g__Lawsonibacter	CTR	2.347	7.85E-07
k__Bacteria.p__Firmicutes.c__Clostridia.o__Clostridiales.f__Clostridiales_unclassified.g__Lawsonibacter.s__Lawsonibacter_asaccharolyticus	CTR	2.347	7.85E-07
k__Bacteria.p__Actinobacteria.c__Coriobacteriia.o__Eggerthellales.f__Eggerthellaceae.g__Adlercreutzia.s__Adlercreutzia_equolifaciens	CTR	2.279	0.00124772
k__Bacteria.p__Actinobacteria.c__Coriobacteriia.o__Eggerthellales.f__Eggerthellaceae.g__Adlercreutzia	CTR	2.276	0.00124772
k__Bacteria.p__Actinobacteria.c__Coriobacteriia.o__Eggerthellales.f__Eggerthellaceae.g__Gordonibacter.s__Gordonibacter_pamelaeae	CTR	2.216	1.58E-05
k__Bacteria.p__Actinobacteria.c__Coriobacteriia.o__Eggerthellales.f__Eggerthellaceae.g__Gordonibacter	CTR	2.216	1.58E-05

Table S5. (continued)

Taxon	Disease	LDA Score	p-val
k__Bacteria.p__Firmicutes.c__Clostridia.o__Clostridiales.f__Lachnospiraceae.g__Blautia.s__Ruminococcus_gnavus	HCC	4.122	0.00980002
k__Bacteria.p__Actinobacteria.c__Actinobacteria.o__Bifidobacteriales.f__Bifidobacteriaceae.g__Bifidobacterium.s__Bifidobacterium_longum	HCC	3.663	0.00203375
k__Bacteria.p__Firmicutes.c__Bacilli.o__Lactobacillales.f__Lactobacillaceae.g__Lactobacillus.s__Lactobacillus_salivarius	HCC	3.656	2.78E-05
k__Bacteria.p__Firmicutes.c__Bacilli.o__Lactobacillales.f__Streptococcaceae.g__Streptococcus.s__Streptococcus_anginosus_group	HCC	3.274	5.21E-05
k__Bacteria.p__Bacteroidetes.c__Bacteroidia.o__Bacteroidales.f__Bacteroidaceae.g__Bacteroides.s__Bacteroides_finegoldii	HCC	3.208	0.00144418
k__Bacteria.p__Firmicutes.c__Bacilli.o__Lactobacillales.f__Lactobacillaceae.g__Lactobacillus.s__Lactobacillus_mucosae	HCC	3.133	2.88E-05
k__Bacteria.p__Firmicutes.c__Bacilli.o__Lactobacillales.f__Streptococcaceae.g__Streptococcus.s__Streptococcus_vestibularis	HCC	3.123	0.00024527
k__Bacteria.p__Firmicutes.c__Bacilli.o__Lactobacillales.f__Lactobacillaceae.g__Lactobacillus.s__Lactobacillus_fermentum	HCC	3.060	5.90E-05
k__Bacteria.p__Firmicutes.c__Clostridia.o__Clostridiales.f__Ruminococcaceae.g__Flavonifractor	HCC	2.977	5.72E-05
k__Bacteria.p__Firmicutes.c__Clostridia.o__Clostridiales.f__Ruminococcaceae.g__Flavonifractor.s__Flavonifractor_plautii	HCC	2.974	5.72E-05
k__Bacteria.p__Firmicutes.c__Bacilli.o__Lactobacillales.f__Streptococcaceae.g__Streptococcus.s__Streptococcus_oralis	HCC	2.957	0.00015415
k__Bacteria.p__Firmicutes.c__Bacilli.o__Lactobacillales.f__Streptococcaceae.g__Streptococcus.s__Streptococcus_gordonii	HCC	2.803	4.23E-06
k__Bacteria.p__Firmicutes.c__Clostridia.o__Clostridiales.f__Clostridiaceae.g__Clostridium.s__Clostridium_sp_CAG_299	HCC	2.738	0.00039514

Abbreviations

iCCA: intrahepatic cholangiocarcinoma
HCC: hepatocellular carcinoma
CTR: healthy control

Table S6. Mean sequencing coverage (sequencing depths in X) of all fecal microbiome samples.

	Species	Mean Sequencing Coverage (Depth in X)		
		CTR	HCC	iCCA
iCCA-specific species	*Veillonella atypica*	1	4	28
	Veillonella parvula	1	4	11
	Streptococcus parasanguinis	3	17	21
HCC-specific species	*Ruminococcus gnavus*	13	24	6
	Ligilactobacillus salivarius	1	15	7
	Bacteroides finegoldii	43	31	21
CTR-specific species	*Roseburia intestinalis*	16	13	7
	Anaerostipes hadrus	19	9	7
	Bacteroides uniformis	23	17	5

Abbreviations
iCCA: intrahepatic cholangiocarcinoma
HCC: hepatocellular carcinoma
CTR: healthy control

Table S7. Representative and/or complete genome names, accession numbers and assembly levels used in sequence alignment.

Disease Group	Species	Genome Name	Strain name	RefSeq Accession Number	Assembly Level	Representative Genome
iCCA	*Veillonella atypica*	ASM208276v1	OK5	NZ_CP020566.1	Complete Genome	NO
	Veillonella parvula	48903_D01	NCTC 11810	NZ_LT906445.1	Complete Genome	YES
	Streptococcus parasanguinis	ASM107115v1	451_SPAR	NZ_JVGP01000013.1	Scaffold	YES
HCC	*Ruminococcus gnavus*	ASM983137v1	ATCC 29149	NZ_CP027002.1	Complete Genome	YES
	Ligilactobacillus salivarius	ASM90009461v1	LPM01	NZ_LT604074.1	Complete Genome	YES
	Bacteroides finegoldii	ASM857139v1	BIOML-A8	GCFA_008571395.1	Scaffold	YES
CTR	*Roseburia intestinalis*	GCA_900537995.1	L1-82	NZ_LR027880.1	Complete Genome	YES
	Anaerostipes hadrus	ASM21069v1	SSC/2	NC_021016.1	Complete Genome	YES
	Bacteroides uniformis	ASM1829216v1	CL03T12 C37	NZ_CP072255.1	Complete Genome	YES

Abbreviations
N/A: not applicable
ns: not significant
FDR: false discovery rate
HCC: hepatocellular carcinoma
iCCA: intrahepatic cholangiocarcinoma
CTR: healthy control
MAG: metagenome assembled genome

Table S8. Metagenome-assembled genomes (MAGs) that matched LDA results.

	Species	Bin Id	# unique markers (of 43)	# multi-copy	GC	Genome size (Mbp)	Gene count	Coding density	Completeness	Contamination	Strain heterogeneity
iCCA	*V. atypica*	MAG286	22	15	39.3430377	1.358811	1396	0.879725	56.76	6.69	50
		MAG320	40	0	39.3722664	1.862765	1791	0.8881217	98.88	1.25	100
		MAG408	27	3	39.9849966	1.469771	1511	0.8847474	71.14	8.66	77.14
	S. parasanguinis	MAG311	26	0	43.7372931	1.194853	1260	0.9148205	65.87	0.52	33.33
		MAG424	31	1	42.4481831	1.886006	1972	0.8688626	69.85	7.05	36.54
		MAG545	32	0	43.0693038	1.903357	1929	0.9045161	90.49	7.93	28.89
	V. parvula	MAG024	26	8	40.6112444	1.303528	1238	0.9256924	56.82	5.04	37.14
HCC	*R. gnavus*	MAG465	43	0	42.8814576	3.255403	3094	0.9088168	99.42	0	0
	L. salivarius	MAG099	43	0	33.2484479	1.898512	1998	0.894599	94.64	2.62	0
	B. thetaiotaomicron	MAG469	42	0	42.7568246	5.734041	4480	0.908208	97.1	1.29	76.47
CTR	*R. intestinalis*	MAG560	29	0	43.0488617	3.096373	2728	0.8946328	92.75	0.85	0
	A. hadrus	MAG179	43	0	37.2641067	2.636512	2487	0.8991436	99.33	1.34	0
		MAG335	43	0	37.4663038	2.665872	2532	0.9111559	97.99	2.01	0
	B. uniformis	MAG353	40	1	46.4168652	4.398963	3620	0.9116601	98.73	2.02	76.92

Abbreviations
iCCA: intrahepatic cholangiocarcinoma
HCC: hepatocellular carcinoma
CTR: healthy control

Table S9. Pairwise FDR-adjusted p-values from Wilcoxon Rank Sum test between disease conditions of MAGs.

			FDR-adjusted Wilcoxon Rank Sum p-value		
			CTR		HCC
**Species-specific to**	**Species**	**MAG**	**iCCA**	**HCC**	**iCCA**
iCCA	V. atypica	MAG286	0.051	0.079	0.557
		**MAG320**	**7.68E-06**	**0.029**	**0.003**
		**MAG408**	**5.58E-06**	**0.019**	**0.005**
	V. parvula	**MAG024**	**4.08E-05**	0.201	**4.08E-05**
	S. parasanguinis	**MAG311**	**0.007**	**0.008**	0.557
		MAG424	1	1	1
		**MAG545**	**0.007**	**0.007**	0.411
HCC	R. gnavus	**MAG465**	**0.002**	0.496	**0.008**
	L. salivarius	**MAG099**	**0.021**	**0.007**	0.606
	B. thetaiotaomicron	MAG469	1	1	1
CTR	R. intestinalis	**MAG560**	**0.007**	**0.008**	0.511
	A. hadrus	**MAG179**	**0.003**	**0.003**	0.851
		**MAG335**	**0.007**	**0.008**	0.526
	B. uniformis	**MAG353**	**0.00025**	0.297	**0.016**

Abbreviations
N/A: not applicable, ns: not significant, FDR: false discovery rate
iCCA: intrahepatic cholangiocarcinoma
HCC: hepatocellular carcinoma
CTR: healthy control
MAG: metagenome assembled genome

Table S10. List of enriched microbial and serum metabolic pathways.

	**Microbial metabolic pathways**				**Serum metabolic pathways**			
	**Pathway Name**	**Pathway ID (source)**	**Full Pathway Name**	**q-value**	**Pathway Name**	**Pathway ID (KEGG Orthology)**	**Full Pathway Name**	**MSEA FDR-adj p-value**
**Enriched in HCC**	**M1.1**	PHOSLIPSYN-PWY (MetaCyc)	superpathway of phospholipid biosynthesis I (bacteria)	0.0646	**S1**	KO00564	Glycerophospholipid metabolism	0.0001
	**M1.2**	PWY4FS-7 (MetaCyc)	phosphatidylglycerol biosynthesis I	0.0646				
	**M1.3**	PWY4FS-8 (MetaCyc)	phosphatidylglycerol biosynthesis II	0.0646				
	**M2**	THISYNARA-PWY (MetaCyc)	superpathway of thiamine diphosphate biosynthesis III (eukaryotes)	0.0646	**S2**	KO00730	Thiamine metabolism	0.071
**Enriched in iCCA**	**M3.1**	PWY-724 (MetaCyc)	superpathway of L-lysine, L-threonine and L-methionine biosynthesis II	0.0646	**S3**	KO00260	Glycine, serine and threonine metabolism	0.024
	**M3.2**	ILEUSYN-PWY (MetaCyc)	L-isoleucine biosynthesis I (from threonine)	0.0646				
	**M3.3**	THRESYN-PWY (MetaCyc)	Superpathway of L-threonine biosynthesis	0.0646				
	**M4**	HISTSYN-PWY (MetaCyc)	L-histidine biosynthesis	0.0646	**S4**	KO00340	Histidine metabolism	0.016
	**M5**	PWY-5686 (HumanCyc)	UMP biosynthesis	0.0646	**S5**	KO00250	Alanine, aspartate and glutamate metabolism	0.016
	**M6**	PWY-1042 (BioCyc)	Glycolysis IV (plant cytosol)	0.0646	**S6**	KO00010	Glycolysis / Gluconeogenesis	0.055

Table S11. Analysis of covariance (ANCOVA) using the metabolite levels as dependent variables with disease status (iCCA and HCC) and individual risk factors as covariates

Risk factors	Body Mass Index			Agriculture jobs			Smoking status			Currently Smoking			Alcohol consumption		
	ANCOVA adjusted p-value			ANCOVA adjusted p-value			ANCOVA adjusted p-value			ANCOVA adjusted p-value			ANCOVA adjusted p-value		
**Metabolites**	Model*	Disease status#	Covariate †	Model*	Disease status#	Covariate †	Model*	Disease status#	Covariate †	Model*	Disease status#	Covariate †	Model*	Disease status#	Covariate †
stearoyl-arachidonoyl-glycerophosphoethanolamine (1)*	**2.32E-06**	**1.92E-06**	0.630	**2.57E-06**	**6.58E-06**	0.977	**3.85E-06**	**4.13E-06**	0.836	**5.81E-06**	**5.91E-06**	0.979	**3.93E-06**	**1.21E-05**	0.875
1-linoleoylglycerophospho ethanolamine*	**0.002**	**0.002**	0.630	**0.002**	**0.004**	0.955	**0.002**	**0.002**	0.241	**0.002**	**0.001**	0.083	**0.002**	**0.003**	0.942
choline phosphate	**1.43E-06**	**1.92E-06**	0.630	**1.38E-06**	**6.58E-06**	0.321	**1.43E-06**	**1.13E-06**	0.165	**3.13E-06**	**3.20E-06**	0.979	**1.44E-06**	**1.31E-06**	0.942
stearoyl-arachidonoyl-glycerophosphocholine (2)*	**0.003**	**0.003**	0.825	**0.002**	**0.011**	0.258	**0.002**	**0.003**	0.609	**0.002**	**0.002**	0.979	**0.004**	**0.004**	0.949
palmitoyl-oleoyl-glycerophosphocholine (1)*	**4.09E-04**	**0.001**	0.763	**4.03E-04**	**0.001**	0.977	**4.93E-04**	**0.001**	0.836	**0.001**	**0.001**	0.979	**0.000**	**0.001**	0.942
palmitoyl-linoleoyl-glycerophosphocholine (2)*	**0.002**	**0.002**	0.825	**0.001**	**0.008**	0.059	**0.002**	**0.002**	0.836	**0.002**	**0.002**	0.693	**0.001**	**0.002**	0.875
palmitoyl-arachidonoyl-glycerophosphocholine (2)*	**0.002**	**0.002**	0.655	**0.002**	**0.005**	0.732	**0.002**	**0.002**	0.836	**0.002**	**0.002**	0.979	**0.003**	**0.003**	0.949
2-palmitoleoyl glycerophosphocholine*	**0.003**	**0.003**	0.763	**0.002**	**0.011**	0.118	**0.003**	**0.003**	0.985	**0.003**	**0.003**	0.979	**0.003**	**0.003**	0.949
1-arachidonoyl glycerophosphocholine (20:4n6)*	**0.007**	**0.007**	0.655	**0.007**	**0.016**	0.693	**0.008**	**0.009**	0.985	**0.009**	**0.009**	0.979	**0.009**	**0.007**	0.942
cysteine	0.146	0.158	0.630	0.148	0.192	0.955	0.161	0.166	0.836	0.162	0.146	0.693	0.128	0.137	0.949
threonine	0.065	0.086	0.655	0.066	0.086	0.955	0.068	0.078	0.154	0.086	0.078	0.693	0.078	0.137	0.743
dimethylglycine	**0.020**	**0.030**	0.630	**0.021**	**0.018**	0.864	**0.017**	**0.017**	0.985	**0.015**	**0.017**	0.979	**0.029**	**0.020**	0.875
histidine	**0.002**	**0.002**	0.630	**0.002**	**0.002**	0.821	**0.002**	**0.002**	0.836	**0.002**	**0.002**	0.693	**0.002**	**0.003**	0.942
glutamate	**0.005**	**0.006**	0.756	**0.005**	**0.011**	0.864	**0.008**	**0.008**	0.836	**0.007**	**0.007**	0.979	**0.003**	**0.003**	0.949
aspartate	**0.013**	**0.014**	0.756	**0.013**	**0.017**	0.955	**0.017**	**0.017**	0.985	**0.013**	**0.014**	0.979	**0.008**	**0.009**	0.949
glucose	**0.020**	**0.014**	0.630	**0.021**	**0.028**	0.955	**0.021**	**0.020**	0.985	**0.023**	**0.022**	0.979	**0.035**	0.057	0.875
lactate	0.153	0.113	0.630	0.152	0.287	0.321	0.184	0.184	0.985	0.182	0.176	0.979	0.138	0.251	0.743
pyruvate	0.097	0.067	0.630	0.091	0.269	0.063	0.087	0.083	0.836	0.113	0.111	0.979	0.115	0.137	0.942

Table S11. (continued)

**Risk factors**	**HBV status**			**HCV status**			**History of OV**			**Family history of cancer**			**History of diabetes**		
	ANCOVA adjusted p-value			ANCOVA adjusted p-value			ANCOVA adjusted p-value			ANCOVA adjusted p-value			ANCOVA adjusted p-value		
**Metabolites**	Model*	Disease status#	Covariate †	Model*	Disease status#	Covariate †	Model*	Disease status#	Covariate †	Model*	Disease status#	Covariate †	Model*	Disease status#	Covariate †
stearoyl-arachidonoyl-glycerophosphoethanolamine (1)*	**8.14E-05**	**3.18E-04**	0.998	**2.56E-06**	**6.35E-05**	0.675	**2.67E-05**	**2.91E-05**	0.895	**3.11E-06**	**4.04E-06**	0.910	**3.13E-06**	**4.34E-06**	0.548
1-linoleoylglycerophospho ethanolamine*	**0.002**	**0.028**	0.645	**0.002**	**0.006**	0.675	**0.012**	**0.014**	0.683	**0.001**	**0.001**	0.238	**0.002**	**0.002**	0.320
choline phosphate	**3.08E-05**	**1.22E-04**	0.998	**1.42E-06**	**1.72E-06**	0.476	**2.67E-05**	**2.91E-05**	0.683	**1.76E-06**	**2.77E-06**	0.910	**1.23E-05**	**8.65E-06**	0.343
stearoyl-arachidonoyl-glycerophosphocholine (2)*	**0.002**	**0.002**	0.936	**0.002**	**0.026**	0.149	**0.013**	**0.016**	0.457	**0.005**	**0.007**	0.910	**0.001**	**0.001**	0.343
palmitoyl-oleoyl-glycerophosphocholine (1)*	**0.001**	**0.000**	0.936	**3.68E-04**	**0.006**	0.503	**0.001**	**0.001**	0.895	**0.001**	**0.001**	0.910	**8.69E-05**	**4.47E-05**	0.103
palmitoyl-linoleoyl-glycerophosphocholine (2)*	**0.002**	**0.025**	0.936	**0.002**	**0.007**	0.891	**0.007**	**0.009**	0.067	**0.001**	**0.001**	0.910	**0.002**	**0.003**	0.268
palmitoyl-arachidonoyl-glycerophosphocholine (2)*	**0.002**	**0.014**	0.998	**0.002**	**0.016**	0.269	**0.004**	**0.006**	0.271	**0.003**	**0.005**	0.910	**0.002**	**0.002**	0.916
2-palmitoleoyl glycerophosphocholine*	**0.002**	**0.004**	0.998	**0.003**	**0.017**	0.675	**0.008**	**0.009**	0.911	**0.004**	**0.006**	0.910	**0.003**	**0.002**	**0.008**
1-arachidonoyl glycerophosphocholine (20:4n6)*	**0.008**	**0.026**	0.998	**0.006**	0.104	0.149	**0.004**	**0.006**	0.683	**0.011**	**0.008**	0.910	**0.011**	**0.008**	0.268
cysteine	0.130	0.070	0.936	0.150	0.200	0.946	0.299	0.309	0.928	0.150	0.151	0.910	0.141	0.163	0.426
threonine	0.064	0.165	0.998	0.064	0.186	0.503	0.074	0.082	0.683	0.097	0.066	0.910	0.076	0.089	0.573
dimethylglycine	**0.024**	0.061	0.936	**0.019**	0.186	0.126	**0.042**	**0.047**	0.895	**0.046**	**0.034**	0.910	**0.011**	**0.013**	0.426
histidine	**0.002**	**0.009**	0.998	**0.002**	**0.006**	0.946	**0.004**	**0.006**	0.683	**0.003**	**0.004**	0.910	**0.002**	**0.002**	0.295
glutamate	**0.003**	**0.007**	0.936	**0.005**	**0.009**	0.675	**0.042**	**0.045**	0.922	**0.005**	**0.007**	0.910	**0.003**	**0.003**	0.573
aspartate	**0.012**	**0.013**	0.936	**0.012**	**0.017**	0.629	0.056	0.055	0.895	**0.013**	**0.018**	0.910	**0.009**	**0.007**	0.343
glucose	0.100	0.285	0.936	**0.020**	**0.022**	0.479	**0.022**	**0.026**	0.547	**0.042**	**0.034**	0.910	**0.014**	**0.035**	**1.03E-04**
lactate	0.219	0.455	0.936	0.154	0.081	0.620	0.299	0.336	0.457	0.150	0.177	0.910	0.211	0.230	0.573
pyruvate	0.100	0.147	0.998	0.095	**0.026**	0.315	**0.042**	**0.047**	0.547	0.098	0.074	0.910	0.076	0.097	0.320

Note:
* The 'Model' column is p-value of the whole model that predicts the metabolite outcome value using both disease status and the covariate (risk factor)
# The 'Disease status column is p-value of the disease status variable that predicts the metabolite outcome alone without the covariate (risk factor)
† The 'Covariate' column is p-value of the risk factor that predicts the metabolite outcome alone without the disease status
All the p-values were FDR-adjusted with Benjamini-Hochberg procedure
